# Learning curves, taking instructions, and patient safety: using a theoretical domains framework in an interview study to investigate prescribing errors among trainee doctors

**DOI:** 10.1186/1748-5908-7-86

**Published:** 2012-09-11

**Authors:** Eilidh M Duncan, Jill J Francis, Marie Johnston, Peter Davey, Simon Maxwell, Gerard A McKay, James McLay, Sarah Ross, Cristín Ryan, David J Webb, Christine Bond

**Affiliations:** 1 Health Psychology Group, Health Services Research Unit, University of Aberdeen, Aberdeen, UK; 2 Health Psychology Group, Institute of Applied Health Sciences, University of Aberdeen, Aberdeen, UK; 3 Division of Population Health Sciences, University of Dundee, Dundee, UK; 4 Clinical Pharmacology Unit, University of Edinburgh, Edinburgh, UK; 5 Department of Clinical Pharmacology and Therapeutics, Glasgow Royal Infirmary, Glasgow, UK; 6 Division of Applied Health Sciences, University of Aberdeen, Aberdeen, UK; 7 Division of Medical and Dental Education, University of Aberdeen, Aberdeen, UK; 8 School of Pharmacy, Queen’s University, Belfast, Northern Ireland; 9 Centre of Academic Primary Care, University of Aberdeen, Aberdeen, UK

**Keywords:** Prescribing, Patient safety, Theoretical domains framework

## Abstract

**Background:**

Prescribing errors are a major source of morbidity and mortality and represent a significant patient safety concern. Evidence suggests that trainee doctors are responsible for most prescribing errors. Understanding the factors that influence prescribing behavior may lead to effective interventions to reduce errors. Existing investigations of prescribing errors have been based on Human Error Theory but not on other relevant behavioral theories. The aim of this study was to apply a broad theory-based approach using the Theoretical Domains Framework (TDF) to investigate prescribing in the hospital context among a sample of trainee doctors.

**Method:**

Semistructured interviews, based on 12 theoretical domains, were conducted with 22 trainee doctors to explore views, opinions, and experiences of prescribing and prescribing errors. Content analysis was conducted, followed by applying relevance criteria and a novel stage of critical appraisal, to identify which theoretical domains could be targeted in interventions to improve prescribing.

**Results:**

Seven theoretical domains met the criteria of relevance: “social professional role and identity,” “environmental context and resources,” “social influences,” “knowledge,” “skills,” “memory, attention, and decision making,” and “behavioral regulation.” From critical appraisal of the interview data, “beliefs about consequences” and “beliefs about capabilities” were also identified as potentially important domains. Interrelationships between domains were evident. Additionally, the data supported theoretical elaboration of the domain behavioral regulation.

**Conclusions:**

In this investigation of hospital-based prescribing, participants’ attributions about causes of errors were used to identify domains that could be targeted in interventions to improve prescribing. In a departure from previous TDF practice, critical appraisal was used to identify additional domains that should also be targeted, despite participants’ perceptions that they were not relevant to prescribing errors. These were beliefs about consequences and beliefs about capabilities. Specifically, in the light of the documented high error rate, beliefs that prescribing errors were not likely to have consequences for patients and that trainee doctors are capable of prescribing without error should also be targeted in an intervention. This study is the first to suggest critical appraisal for domain identification and to use interview data to propose theoretical elaborations and interrelationships between domains.

## Background

Promoting safe medication use is a priority for all healthcare systems and is the focus of initiatives such as the United States’ 100,000 Lives Campaign [1], World Health Organization (WHO) Patient Safety Programme [2], and WHO Curriculum Guide for medical schools [3]. In the United Kingdom, medication incidents are the third most common cause of patient safety events within the National Health Service (NHS), with more than 80,000 reported annually to the National Patient Safety Agency (NPSA). In 2001, the UK Audit Commission’s seminal report, *A Spoonful of Sugar*[4], reported that approximately 1000 patient deaths per year are due to medication errors or adverse drug reactions. In 2007, the NPSA received 100 medication incident reports of death and severe harm [5], of which prescribing errors accounted for 32%. There is little evidence to suggest that these figures have improved. Concerns about the prescribing skills of doctors in training have been raised [5], and doctors in the first two years of postgraduate training (foundation doctors) have been reported to be responsible for the majority (90%) of prescribing errors [6]. Interventions to reduce errors are urgently required.

An understanding of the factors underlying prescribing errors is an important first step in the development of strategies to address this problem. A systematic review of the literature [7], which investigated the causes of prescribing errors using Reason’s Human Error Theory [8], concluded that errors were mainly “mistakes” (*i.e.*, an intended action that goes wrong), with underlying contributing factors of stress, fatigue, high workload, lack of experience or training, and poor communication reported. Dornan *et al.* undertook a large study in England that reported similar results [9]. Franklin *et al.* identified additional themes, such as lack of feedback, lack of documentation of prescribing decisions, a focus on the drug but not on dose and frequency, poor quality of medication information at hospital admission, and a lack of willingness by doctors to challenge senior decisions [6]. In general, only a few have been theoretically based, and there is still insufficient understanding to develop appropriate interventions.

The 2008 Medical Research Council guidance for development of complex interventions [10] proposes that a key task is to develop an understanding of the likely processes of change, utilizing evidence and theory. The aim of this study was to use a theoretical approach to establish an evidence base that may facilitate an understanding of the processes associated with prescribing behavior among doctors in their first two years of training. The Theoretical Domains Framework (TDF [11]) from health psychology provides the basis for such an approach, ensuring that a wide range of possible theoretical explanations for the behaviors is considered. Built from 33 behavioral theories, it proposes that determinants of healthcare professionals’ behavior cluster into 12 domains (*e.g.*, “social influences,” “behavioral regulation,” “social/professional role and identity”). The framework is not a theory, as it does not propose relationships between its elements, but it has been used to identify barriers to quality improvement in healthcare in order to develop interventions [12]. Evidence suggests that TDF-based interviews may prompt respondents to identify barriers that they would not otherwise report [13]. This article is one in a series of articles documenting the development and use of the TDF to advance the science of implementation research. The series’ introductory article [14] provides an overview and critique of the framework. In behavioral research, the preliminary step in the process of developing interventions is to specify the behavior under investigation [15]. For example, in predictive questionnaire studies applying the theory of planned behavior, [16] a recommended approach is to specify the behavior using the Target, Action, Context, and Time (TACT) principle [17] (*i.e.*, specifying doing what, to whom, when, and where). However, this approach is often regarded as restrictive. For example, the predictors of a physician’s intentions to prescribe a specific dose of a specific drug when Mrs. X comes to discuss her test results next Tuesday may not generalize to other patients or other clinical situations. Furthermore, many clinical actions are complex, so specifying behavior is not always straightforward. For example, a study identifying the key behaviors of best practice when disclosing a diagnosis of dementia used literature review, interview, consensus process, and content analysis methods to identify eight categories and 220 component behaviors [18]. There is thus a balance to be judged between precise specification of behaviors and maximizing the clinical usefulness and generalizability of results.

Prescribing guidelines acknowledge the complexity of the behavior and note that prescribing occurs within hierarchically structured healthcare teams, yet also demands a high level of personal responsibility [19]. In this study, we chose to specify the target behavior of prescribing in broad terms based on the following definition: a clinically meaningful prescribing error occurs when, “as a result of a prescribing decision or prescription-writing process, there is an unintentional significant (1) reduction in the probability of treatment being timely and effective or (2) increase in the risk of harm when compared with generally accepted practice” [20,21]. As suggested by this definition, prescribing involves two component processes: decision making and prescription writing [22]. Examples of the errors that may occur in each of the component process are shown in Table 1. For this context, we considered only “primary” prescribing behavior and did not include the rewriting of existing prescriptions.

**Table 1:** Examples of errors occurring in each prescribing component process

**Prescription writing**	**Decision making**
Wrong patient	Incorrect duration
Medication omitted	Medication omitted
Inappropriate abbreviation	Incorrect timing
Illegible	Incorrect frequency
Incomplete prescription	Incorrect route
Missing instructions for use	Incorrect dose
Omission of prescriber signature	Incorrect formulation
Incorrect drug	Medication prescribed without indication
	Contra-indication to medication
	Significant drug-drug interaction
	Duplication of therapy
	Patient allergic to drug prescribed

To date, the TDF has been applied to very specific (*e.g.*, decision making relating to transfusion practice [23]) and more general behaviors (*e.g.*, implementing guidelines [24,25]). By contrast, this study concerns a “complex” behavior, that is, a range of specific actions, any of which could be classified as a prescribing error. Prescribing is a multifaceted behavior occurring within multiple contextual levels in the hospital environment. This study, therefore, applied the TDF to a behavior that is complex (*i.e.*, involving multiple actions) and multilevel (*i.e.*, performed by multiple healthcare professionals within a hierarchical structure). The study addressed the question, which theoretical domains should be targeted by an intervention to improve prescribing practice? A methodological question considered in this study was whether a TDF-based topic guide was able to prompt participants to discuss their beliefs using this general behavioral description.

This interview study formed part of the PROTECT (PRescribing Outcomes for Trainee doctors Engaged in Clinical Training) program of work, which included a national prospective observational study of prescribing errors made by hospital doctors and interviews and questionnaires investigating trainee doctors’ knowledge, attitudes, and experiences of prescribing errors.

## Methods

### Design

Semistructured interviews were conducted with trainee doctors to investigate their views, opinions, and experiences of prescribing and prescribing errors.

### Sample

The sampling frame was all foundation doctors (*i.e.*, doctors within their first two years of postgraduate training) in 11 hospitals across Scotland. Participants were recruited in two ways: (1) by email invitation circulated by NHS Education for Scotland (the board responsible for developing and delivering training to all trainee doctors in Scotland) to all registered foundation doctors in Scotland (793 year-1 trainees and 804 year-2 trainees) and (2) by presentation of the project, together with an invitation to participate, at routine training sessions attended by foundation doctors in two hospitals. Sixty-two participants (3.9% of all foundation doctors) indicated their willingness to be interviewed either by sending an email or by returning a signed consent form. Of these, 22 were purposively sampled on the following characteristics to achieve a diverse sample: year of training, region in which employed, gender, and current clinical specialty.

### Materials

A semistructured topic guide (Additional file 1) was developed based on the TDF [11] to include questions about factors that might influence prescribing. The topic guide incorporated questions relating to the two processes involved in prescribing for patients: decision making and prescription writing. A definition of prescribing error [21] was discussed with participants, and the examples from Table 1 were provided to interviewees to aid a shared understanding. The topic guide was drafted by one researcher (EMD) and then refined by health psychologists with expertise in the TDF (JJF and MJ) and discussed by the research team to check clinical relevance. The questions were then piloted with one senior (SR) and two trainee doctors to assess clarity and focus, and a final version was agreed upon.

### Procedure

Face-to-face interviews were conducted by EMD (a health psychology researcher with interview training and experience) at a time and place to suit the participant. Interview locations included private offices, doctors’ lounges, and hospital and public cafes. Where participants had indicated by email that they wished to take part, written informed consent was obtained immediately prior to conducting the interviews**.** Where participants had indicated in person that they wished to take part, written informed consent was returned by post**.** Participants were encouraged to reflect upon their own experiences of prescribing and were asked to think about an example of a known prescribing error when answering questions. The interviewer used prompts when necessary to encourage further elaboration. Interviews were recorded and transcribed verbatim by an experienced transcriber employed by the University of Aberdeen. The researcher (EMD) then proofread the transcripts, editing where necessary to ensure accuracy. Interviewees were provided with a Continuing Professional Development certificate for participating. The study was approved by the North of Scotland Research Ethics Committee and NHS Grampian Research and Development.

### Analysis

Transcripts were first anonymized and then an extensive familiarization process was carried out. In line with framework analysis [26], an overview of the entire data set was gained in order to set the context for data analysis and to gain a feel for the material as a whole. This approach involves a systematic process of sifting, charting, and sorting material according to key issues. A coding index was developed (by EMD) and was critiqued by a second researcher (JJF). The results of this process are reported below under Familiarization Phase. Following this familiarization process, specific beliefs were identified within the data and coded (by two researchers, EMD and JJF) into domains of the TDF [11] using theory-based content analysis.

Two further steps in the analysis were used to identify domains suitable for targeting in an intervention to improve prescribing. First, criteria were applied to the findings to identify the domains that participants perceived to be relevant (*i.e.*, important barriers to appropriate prescribing). Second, a stage of critical appraisal was performed as described below.

### Application of criteria for relevance

Previous research (*e.g.*, [23]) has used relevance criteria to determine which domains could be potential intervention targets. In line with this previous research, the following criteria were applied:

1. Frequent coding of specific beliefs within a domain,

2. Evidence that participants perceived a potential influence on prescribing without error (*i.e.*, they attributed errors to factors that were coded into the domain).

### Critical appraisal

This study, in a departure from previous TDF-based studies, also included a further step in identifying target domains:

3. Critical appraisal of participant responses within each domain to identify further potential targets for intervention.

Participant responses within domains were considered, along with evidence from published literature and other arms of the PROTECT study, to interrogate whether further targets for intervention existed. The results from each stage of the analysis are presented below.

## Results

Sample characteristics

Twenty-two trainee doctors (15 female and 7 male; mean age 25.4 years; 11 foundation year 1 and 11 foundation year 2) participated in interviews. They worked in a range of medical specialties and geographical regions. In Scotland, training for doctors is split across four deaneries; five participants were recruited from the north, six from the east, four from the southeast, and seven from the west regions. Interviews took between 21 and 84 min (mean interview length 44 min).

### Familiarization phase

The familiarization phase resulted in a coding index with three major themes—learning curves (*e.g.*, the influence of clinical experience on prescribing behavior and the knowledge required to prescribe safely), taking instructions (including the role other people play in influencing prescribing and the responsibilities of multiple professional groups to ensure appropriate prescribing), and discussions around patient safety (the consequences of errors, error-producing situations, and strategies to reduce errors). Within these three major themes, subthemes were evident as outlined below.

• Learning curves

○ Influence of gaining clinical experience: the influence of experience and increased knowledge on prescribing behavior

○ Knowledge required: the medical, drug, and procedural knowledge required to make a safe prescription

• Taking instructions

○ Interprofessional responsibilities: the roles and responsibilities of other medical professionals in preventing errors

○ Influence of other people: the influence of senior colleagues, pharmacists, nurses, and patients on prescribing behavior

○ Influence of medical speciality: the trainee doctors’ prescribing duties and responsibilities varied depending on the medical speciality

• Patient safety

○ Confidence about prescribing without error: feelings about ability to prescribe without making an error

○ Error outcomes: perceptions about what might happen after an error is made

○ Situations associated with errors: error-producing conditions and situations

○ Strategies to reduce errors: possible ways of reducing the chances of making prescribing errors

**Figure 1:**
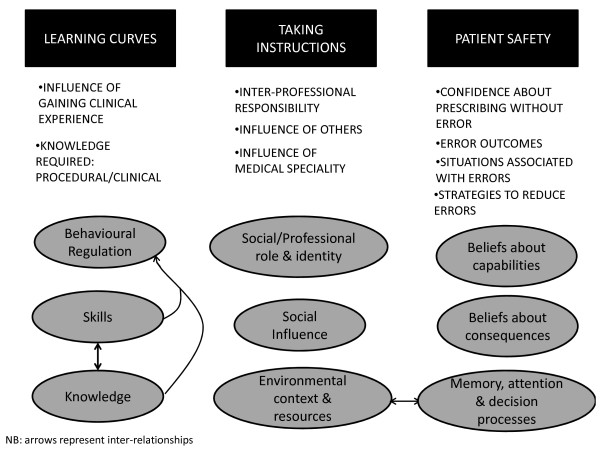
**Relationship between themes identified within the familiarization phase and domain-level coding.** The three main themes identified within the familiarization process are shown at the top of the figure in black boxes; learning curves, taking instructions and patient safety. Each of these themes contained a number of sub-themes shown as bulleted text below the black boxes. The domains-level coding which relates to the learning curves, taking instructions and patient safety themes is shown within the grey circles and arrows represent interrelationships.

The relationships between the thematic codes from the familiarization phase and the domain-level coding (presented in the following section) are shown in Figure 1.

### Content analysis

Directed content analysis [27] was used, with domains from the TDF providing the coding categories. Operational definitions for each domain were produced and critiqued by the research team. Specific beliefs within the interviews were coded into domain categories (Table 2). As displayed in Table 2, individual beliefs were, at times, coded into multiple domains. For example, the domains “knowledge” and “skills” were discussed in this context as being intertwined. Following this stage of content analysis, relevance criteria were applied as per previous TDF studies [23]. The results of this stage of the analysis are reported below.

**Table 2:** Specific beliefs assigned to domains

**Specific beliefs**	**Domain**
My perceptions about my own prescribing have changed with experience.	Skills
Less experience means I may be more likely to make an error.	Knowledge/skills
When I have more experience, I consult reference sources less.	Knowledge/skills
Behavioral regulation	
More experience means I may be more likely to make an error as I may become complacent.	Knowledge/skills
Behavioral regulation	
I need to know professional norms for writing prescriptions in order to prescribe without error.	Knowledge
I need to know about guidelines and protocols to prescribe without error.	Knowledge
I’m not always aware what protocols are in place.	Knowledge
The nurses are good at picking up errors.	Social/professional role & identity
The pharmacist checks my prescriptions for errors (in some wards only).	Social/professional role & identity
Senior colleagues influence my prescribing behaviour.	Social influences
Pharmacists influence my prescribing behaviour.	Social influences
Nurses influence my prescribing behaviour.	Social influences
Everything I write on a prescription will have been told to me by a senior colleague.	Social influences
Support is greater when working on specialist wards.	Social/professional role & identity
I’m confident I don’t make errors when prescribing.	Beliefs about capabilities
If I make an error, it will be picked up by someone else.	Beliefs about consequences
If I make an error, nothing may happen.	Beliefs about consequences
If I make an error, it may not have any effect on the patient.	Beliefs about consequences
If I make a prescribing error, it can cause harm to the patient.	Beliefs about consequences
If I make a prescribing error, it can result in negative outcomes for myself.	Beliefs about consequences
If I am distracted when I’m prescribing, I’m more likely to make an error	Environmental context and resources
Memory, attention, & decision processes	
If I am under time pressure when I’m prescribing, I’m more likely to make an error.	Environmental context and resources
Using reference sources helps me to prescribe without making an error.	Behavioral regulation
Having easily available guidance at the point of prescribing medications would reduce errors.	Behavioral regulation
Having greater pharmacy support would reduce prescribing errors.	Behavioral regulation

### Application of criteria for relevance

Not all domains were found to be relevant to the context of prescribing. Domains that were not identified as relevant were “emotion,” “motivation and goals,” and “nature of the behaviour.” Seven domains were identified as relevant, according to the criteria, and are described below.

### Knowledge

The domain “knowledge” includes “procedural knowledge” and “knowledge about condition/scientific rationale” [11]. When participants discussed the importance of knowledge in prescribing without error, both procedural (*i.e.*, knowledge of the prescribing process) and clinical (*i.e.*, knowledge of the drug, condition, patient details) knowledge were regarded as relevant. These two types of knowledge are discussed further below.

### Procedural knowledge

Knowledge of guidelines and protocols for writing prescriptions was considered to be important but was sometimes lacking.

Just obviously the correct way to fill out a Kardex and certain things you’re not supposed to… Certain abbreviations you can use, like for instance you’re supposed to always write oral or not p.o., but to be honest, loads of people write o and p.o. and all that stuff.—Interviewee 13 (F1; Foundation year 1 doctor).

Participants discussed the challenge of rotating from post to post and noted that not all procedural knowledge was transferable.

I mean it’s a real pain, I mean there’s differences, depending on what, you know, different drugs have different prescription sheets, different wards prescribe things on, on different sheets in different ways … they’ve got different prescription charts. So every time you move onto the next job, you’ve got to sit back and try and work out how they do, how they do it here, you know, what sort of sheet you’re supposed to prescribe it on and that becomes confusing.—Interviewee 6 (F2; Foundation year 2 doctor).

### Clinical knowledge

The clinical knowledge reported as necessary for prescribing treatments for patients included knowledge of drugs, doses, and patients’ conditions and existing medications. The participants reported that, at times, their knowledge fell short of the ideal, and some discussed writing prescriptions without knowing details of the patient.

So in vascular surgery I didn’t have a clue about pretty much any of it, there’s so many diseases and acronyms and sort of thing and drugs and processes that I didn’t know anything about and just had to learn. You know, I mean, I was writing things down on ward round that I didn’t understand, I would write it down and then try and work out what it meant later.—Interviewee 2 (F1).

### Social/professional role and identity

Participants discussed how their prescribing behavior varied according to medical specialty. For example, a number of participants reported that the trainee doctor role in pediatric, neonatal, and renal wards was closely supervised due to the nature of the patients treated and the specialist prescribing required for these patients.

Down here [on a renal ward] it’s very different, because up on general medicine and surgical wards, it tends to be the F1 [foundation year 1 doctor] writing on ward rounds …the F1 that makes changes on the Kardex [drug chart]. But down here that’s taken away, and it’s the ST1s [specialist trainee level 1 doctor] or registrars that make those changes, which is good from our point of view, because of all the specialist drugs.—Interviewee 1 (F1).

In these wards, participants reported that senior colleagues almost always made the prescribing decisions, reviewed trainee doctors’ work regularly, and were easily contactable for advice giving. This led to the perception of some participants that they held little responsibility for prescribing decisions in these wards.

On pediatrics … because obviously the nature of pediatrics, … you can’t afford to make mistakes …. everything you do basically gets reviewed by senior [colleagues]…It makes the job quite easy but …, because it’s not challenging it gets a bit monotonous. But certainly, … there’s very little responsibility for F1s.—Interviewee 2 (F1).

Contrasting views were reported about the role of trainee doctors in surgical rotations. A number reported that surgical rotations included little support from senior colleagues and that contacting others for advice was problematic.

On the medical floor, sometimes you’re not very well supported. The surgical floor as well, especially in general surgery and orthopedics, I know you’re not overly supported by senior [colleagues].—Interviewee 1 (F1).

Interviewees also discussed the demarcation of responsibility for prescribing. Senior colleagues were often reported to be the group making the prescribing decisions, with the prescription writing being done by the trainee doctors. Despite this, participants reported that by signing the prescription, they were taking responsibility for it.

Legally it’s up to us because it’s our signature that’s next to the drug and it is our responsibility to make sure that any drug charts or any prescriptions that we do is free from error.—Interviewee 3 (F2).

Participants also discussed overlapping roles in ensuring prescriptions are error free. Nurses were perceived to be good at identifying errors before they reached the patient and were reported as sharing responsibility for ensuring that prescribing errors did not reach patients.

…if I’ve actually prescribed [the drug] incorrectly and the nurse [has given the drug], I think the nurse is still accountable, because she’s administered the drug…. From what I’m led to believe, if the nurse goes ahead and gives it, she’s equally to blame, and I’ve heard that the nurse is disciplined a lot harder than the doctor is, for some reason.—Interviewee 1 (F1).

Pharmacists were also reported to be checking prescriptions and taking responsibility for noticing errors.

… prescriptions are always checked by the pharmacist on the ward, most errors are picked up that way. They [pharmacists] go through the Kardexes [drug charts], make sure everything is correct. Usually they’re very good at picking up errors.—Interviewee 4 (F1).

### Social influences

Participants discussed the influence that other people had on their prescribing.

There was evidence of two kinds of social influence: informational influence (the influence of other people’s knowledge and skills on own behavior [28]) and injunctive normative influence (the influence of what other people want/expect one to do [29,30]). Senior doctors were seen to be highly influential in influencing prescribing behavior through both the informational and normative pathways.

When you’re on the ward and your senior [colleague] says, “write them up for whatever,” then as the junior you do what you’re told.—Interviewee 5 (F1).

Pharmacists had an informational influence and were viewed as a useful guidance resource.

I felt much more reassured to know that there was a pharmacist around, and they’re very approachable so that really helped.—Interviewee 9 (F1).

Nurses were also reported to be influential both in terms of informational and normative influences; however, a number of participants reported that this influence was treated with caution at times.

They [nurses] are very good at giving advice. Even though nurses are not allowed to prescribe, they will come and tell you “this is what I want you to prescribe” and generally it’s good to follow their advice because they know…. Sometimes the nurse will say “oh are you sure about that?” and then it depends on the seniority of the nurse and the experience of the nurse that you’d … question yourself and say well actually, maybe you are right.—Interviewee 11 (F2).

Also discussed was the influence of patients and patients’ relatives on prescribing, with some reports that patients may influence drug choice or dosage.

…the patient still wasn’t happy so we kind of reached an agreement with him that we’d give him 50% extra dose the first day and then go with the normal dose after that. Sometimes patients just don’t want to take the medication, they’re really against it. Things like pain relief we kind of have to compromise quite often with them.—Interviewee 12 (F1).

However, many of the participants reported that patients’ views may not always be taken into account when prescribing.

Interviewer: What about other people then, maybe the patient, would their views affect the process of prescribing?

Interviewee 4 (F1): If they’ve any allergies to [any drugs], but not specifically … their views I don’t think, at least not that I’ve encountered.

### Environmental context and resources

Participants discussed contextual factors that may make errors more likely to occur. The most discussed factor attributed to errors was distraction (discussed by eight participants). Examples of distractions included phone calls, pager messages, and interruptions from colleagues as shown in the following quote.

You do have such a high workload, you’ve got so much to cram in during the day, so when you’re prescribing… you’ve got nurses asking you questions, you’re trying to remember all the jobs, you’ve got phone calls, answering your bleep [bleeper]…. There are quite a lot of distractions when you’re actually writing the Kardex [drug chart].—Interviewee 17 (F1)

The challenge inherent in handling interruptions from senior colleagues was discussed.

The junior person can’t dismiss their boss and say, “Hold on a minute, I’m busy.” You can’t do that.—Interviewee 6 (F2).

Another factor widely reported to cause errors was the time pressure experienced as a trainee doctor and how this impacted upon capacity to check the prescription.

Sometimes [factors] like time pressures… I think that’s when errors occur, because you don’t always have time, you’re in the middle of a ward round and someone will say, “Just write this patient up for whatever,” and then you move quickly onto the next patient and you can’t always be sort of like looking up the BNF [British National Formulary].—Interviewee 5 (F1).

A related factor discussed by interviewees was the workload expected of trainee doctors.

When you’re overloaded with work and [have] too many things to do at once … when you’ve got too much things to do and one thing slips your mind.—Interviewee 4 (F1).

Issues surrounding the availability of resources, including written and verbal guidance, were also discussed.

I have limited access to the online thing [emergency care summary], so I have to find the time to phone the GP [general practitioner]… it can be a bit of an issue.—Interviewee 7 (F2).

Further situations where errors were perceived to be more likely included working out of hours, rushed hand-overs with colleagues, working in a new ward, and some aspects of prescription chart design.

### Critical appraisal

“Beliefs about capabilities” and “beliefs about consequences” did not fit the criteria for relevant domains. Although information relating to these two domains was frequently mentioned, the participants did not make an explicit link back to their prescribing behavior. Beliefs about own capabilities varied from interviewee to interviewee, but no direct link between this belief and own behavior was made. Similarly, although interviewees were cognizant of the potential consequences of prescribing errors, they did not discuss these beliefs as explicitly influencing their behavior. However, critical reflection of these domains suggested that participants’ lack of an explicit link indicated that these domains may need to be targeted in any intervention effort. There is evidence (including from other arms of the PROTECT study) that prescribing errors occur frequently and that the clinical consequences can be serious. Therefore, the discrepancy between objective evidence and trainee doctors’ beliefs about consequences and their capabilities could itself be an appropriate target for intervention.

### Findings: theoretical lens

During the process of coding beliefs into domains, two points of theoretical significance became apparent. First, participants made explicit associations between some domains. Second, the complexity of the data within the domain “behavioral regulation” suggested that further elaboration of this domain could be helpful. Data relating to these two topics are reported next.

Some themes mapped cleanly onto discrete domains, while others suggested relationships between domains. The domains “knowledge” and “skills”, in this context, co-occurred in the sense that the participants discussed them together.

If you’re prescribing the drugs on a day-to-day basis, you get to know the correct dose and frequency and that sort of thing.—Interviewee 1 (F1).

A lot of the things that we prescribe now are things that you prescribe over and over again. We [learn] over time.—Interviewee 4 (F1).

Participants inferred an influence of knowledge and skills on behavioral regulation (*i.e.*, as knowledge/skill increases, behavioural-regulation strategies such as consulting guidance documents may decrease). They discussed how clinical experience affected their prescribing behavior, reporting that when their levels of knowledge and skills were lower, they were more likely to refer to the British National Formulary (BNF) and ask colleagues for advice to support their prescribing than when knowledge and skills increased.

I would have to look things up in the BNF like the doses for things…[because] I didn’t know off the top of my head or I would double-check things with a senior colleague an awful lot more than I do now.—Interviewee 5 (F1).

I remember at the start I would have to ask every time I was gonna prescribe something. I just wanted to check that it was fine, but now you…do things day to day and you…get used to what you should be prescribing.—Interviewee 12 (F1).

There was a perceived paradoxical influence of experience on the likelihood of making an error; both low and high levels of experience were reported to be associated with a greater chance of making an error. Less-experienced doctors were considered to be inherently more likely to make an error but were also more likely to check information sources to verify their prescribing, whereas with experience, doctors reported feeling more confident in their ability and therefore less likely to consult external sources and hence more likely to make an error.

…with experience, you obviously know more about medications and why you give them, but then, with experience you…may also get…careless…. Careless as in…people might not bother to check with the BNF.—Interviewee 4 (F1)

The domains “environmental context and resources” and “memory, attention, and decision making” appeared to be related. As mentioned previously, participants reported contextual issues, such as distraction and time pressures, as influencing prescribing behavior. Participants considered these contextual issues to influence their cognitive capacity to prescribe without making an error (*i.e.*, by impacting on attentional control and memory).

If they are interrupting you, which quite often happens, then I think that really affects the process of prescribing, and it quite annoys me when people do interrupt you when you’re writing a Kardex [drug chart] because it’s so easy to make a mistake and…I think less mistakes would happen if you could sit in a quiet room and write out the charts quietly rather than on a busy ward with people coming up to you.—Interviewee 10 (F1)

The content of the data relating to the “behavioral regulation” domain suggested that expansion was possible. Behavioral regulation refers to strategies for translating motivation into action, including preparatory steps [11]. Within the “behavioral regulation” domain, multiple levels were evident within the data. Content related to individual, profession-wide, and hospital-wide levels.

### Individual level

All participants reported using some form of guidance to inform their prescribing at least some of the time. Guidance sources included local formularies and national (*e.g.*, the BNF) and international protocols (*e.g.*, WHO pain ladder [31]) and were reported to be beneficial to practice. Some participants reported that consulting guidelines could be time consuming.

I think I was prepared to look everything up, even if it took twice as long, just to make sure that I was writing it correct.—Interviewee 7 (F2)

A number of participants reported being unaware of guidelines and reported prescribing treatments without referring to guidance documents.

I just, it got to the point that I was like, “OK, I can’t really get away with not knowing, just prescribing blindly,” let’s ask why I’m doing this and she showed me the whole protocol. I didn’t even know these protocols existed.—Interviewee 2 (F1)

Senior colleagues were reported to go against protocols, at times, when prescribing, and trainee doctors were reported to be more likely to follow protocols.

So, yes there are protocols but…a lot of times those protocols are ignored due to experience of the consultant…or…lack of caring by the consultant about these new protocols. Sometimes it does seem [like there is a] “consultant knows best” sort of attitude, and damn the protocols, it doesn’t really matter… I’m aware [of] these protocols, I think certainly the juniors are more aware that these protocols exist, the further up you go [seniority of colleagues], the less they seem to matter.—Interviewee 2 (F1)

The participants described a range of strategies they used to improve their prescribing practice. These included being aware of their own limitations and checking their prescriptions.

I think you can always reduce it [the likelihood of making an error]…[by] working within the limitations of your knowledge and by double-checking everything.—Interviewee 12 (F1)

Further strategies used by participants included making up lists of commonly used drugs and doses to carry with them while they worked.

I walked around with a piece of paper that my handover F1 [wrote for me] because I was on a night shift, I was on my own at night…. [The piece of paper included] basic analgesia step-up ladder and basic things to use for a patient who wants a sedative, you know, that was very, very helpful actually and that was just something one of my friends put together for me and I walk around with that in my pocket and it was like my little bible when I go on nights.—Interviewee 14 (F2)

### Profession-wide level

The trainee doctors interviewed reported a need for more teaching before postgraduate training commences, including further teaching on pharmacology and greater training in prescribing.

Personally, I think the medical school didn’t give us enough pharmacology education and knowledge about the different drugs. I think, having teaching, as well, as a junior doctor is really important to…continuing education.— Interviewee 10 (F2)

### Hospital-wide level

A number of hospital-level strategies to reduce prescribing errors were suggested. These included having easily available guidance material at the point of prescribing, such as access to the Emergency Care Summary (an electronic record of patients’ information about any medicines prescribed by the patient’s general practitioner and about any adverse drug reactions that the general practitioner knows about), posters on the wall, and laminated sheets providing information of usual prescriptions.

Participants also reported that greater pharmacy support would be beneficial in reducing errors made.

…the sort of set up that we had in my first F1 job…you would always have a pharmacist who would check over [prescriptions] and were there to consult for advice.—Interviewee 3 (F2)

A further error-reduction strategy proposed by the participants interviewed was better feedback about errors they had made.

…having an environment where if you do make a mistake someone should explain it to you, it’s good to get feedback.—Interviewee 7 (F2)

As a further check of the coverage of the TDF-based interview guide, participants were asked towards the end of the interview, “Is there anything else that we’ve not covered that you feel is important? Is there anything else you’d like to say?” No new data were generated from this prompt, suggesting that the TDF-based interview guide prompted appropriate coverage of this topic.

## Discussion

The TDF has facilitated the investigation of a complex behavior with important implications for one aspect of patient safety: prescribing without error by trainee doctors. The thematic content of the interviews covered three main areas: learning curves associated with commencing the first years of postgraduate training; taking instructions and other influences of colleagues on their prescribing; and issues around patient safety, including strategies to reduce prescribing errors. Other theoretical frameworks, notably Human Error Theory [8], have been used to explore trainee doctors’ views about prescribing errors [6,32]. Applying the TDF has added to this field of investigation by prompting consideration of a wider range of possible precursors of these errors.

Using previously published criteria [23], seven theoretical domains were identified as relevant for prescribing behavior: “knowledge”; “skills”; “behavioral regulation”; “environmental context and resources”; “social influences”; “social/professional role and identity”; and “memory, attention, and decision processes.” These results suggest that interventions designed to improve the prescribing practice of trainee doctors could include a number of behaviour-change techniques that target these domains [33] (as shown in Table 3). In addition, these findings suggest that environmental changes may facilitate improved prescribing behavior (*e.g.*, greater access to sources of information, reducing distractions and interruptions at time of prescribing, and redesigning prescription charts).

**Table 3:** **Behaviour-change techniques suggested by results (based on**[35])

**Domain(s)**	**Example behaviour-change technique**
Social/professional role and identity	Social processes of encouragement, pressure, and support
Social influences	Modeling/demonstration of prescribing behavior by other colleagues
Knowledge	Information regarding prescribing errors and the outcomes of errors
Skills	Rehearsal of appropriate prescribing behaviors
Environmental context and resources	Environmental changes to facilitate prescribing
Memory, attention, and decision processes	Self-monitoring of prescribing behavior
Behavioral regulation	Prompts, triggers, and cues
Beliefs about capabilities	Feedback about prescribing errors made
Beliefs about consequences	Persuasive communication

In a departure from previous methods of applying the TDF, a critical appraisal stage was conducted in order to further interrogate the findings and to decide whether more domains should be targeted in an intervention to improve prescribing errors. “Beliefs about consequences” and “beliefs about capabilities” were discussed by participants but did not meet the criteria for relevance as no explicit link was made in terms of these domains influencing their prescribing behavior. However, the critical appraisal stage suggested that these two domains should still be included. This finding highlights that further interrogation of the criteria for determining which domains should be the basis of an intervention would be beneficial. It is questionable whether we can rely on participants themselves to identify what needs to be changed in order to improve prescribing behavior. We know from previous research that individuals are biased in their views about the causes of their own behavior and tend to attribute failures to external (environment or other people) rather than internal (ability, effort) factors [34,35]. Furthermore, there is evidence that prescribing errors occur frequently and that the clinical consequences can be serious (PROTECT manuscript in preparation). Therefore, the discrepancy between objective evidence and trainee doctors’ beliefs about consequences and their capabilities could itself be an appropriate target for intervention. The results of this study suggest that careful consideration needs to be given to critically appraise the data when deciding which domains could be taken forward as targets for intervention.

There are a number of limitations of this study. The response rate to the invitation to participate was low, due possibly to the sensitive nature of prescribing errors. As reported by those interviewed for this study, their time in training is inherently busy, which may have impacted upon response rates. However, despite this low response rate, participant diversity was evident for a range of target variables (*e.g.*, year of training, clinical specialties experienced, region trained). A further possible limitation is that, despite efforts to ensure interview privacy, a number of interviews had to be conducted within public places, which may have led to participants feeling less able to divulge certain information. Furthermore, the approach to qualitative methods and analysis applied in this study represents the disciplinary perspectives of health psychology and, as such, may not necessarily be shared by all other disciplines. Finally, the data presented here relate to participants’ attributions of the influences on their prescribing behavior, and so these findings do not demonstrate actual causes.

Despite these limitations, this research can offer a number of insights into both the use of the TDF to explore a complex clinical behavior and potential strategies for interventions to improve prescribing practice. This study is the first to use this theoretical approach to understand prescribing errors, and thereby adds to the literature that is often based on Human Error Theory alone. Using the TDF in this way allows for the findings to be linked to theory and subsequent targeting of appropriate behaviour-change techniques. Furthermore, the TDF ensured that a wide range of influences on prescribing behavior was considered, rather than the restricted set of influences that may be explored when research is limited to individual theories of behavior.

Although this study was not designed to evaluate the TDF, the results suggest that greater consideration of the interrelationships between theoretical domains may also be warranted. The original TDF did not attempt to identify relationships between domains, although several theories on which it is based do specify relationships between constructs. In this application of the framework to prescribing behavior, the “knowledge” and “skills” domains were often discussed together and were associated with an impact upon “behavioral regulation.” This result links with previous research [36] investigating skill acquisition in nurses that found that new graduates were heavily reliant on guidelines, with this reliance reducing as their experience increased. An additional interrelationship was found: “environmental context and resources” (*e.g.*, the distractions arising from a busy clinical environment) was discussed in association with “memory, attention, and decision processes” (*e.g.*, distractions influenced ability to concentrate on prescribing decisions). The results also suggest the potential for greater elaboration of the “behavioral regulation” domain, highlighting the importance of interventions targeting multiple levels of healthcare systems in accordance with Ferlie and Shortell’s guidance [37]. These findings suggest that there is potential for developing the theoretical links between the domains.

## Conclusions

The first years of postgraduate medical training in hospitals present a challenge both for the doctors involved and for patient safety. In this investigation of hospital-based prescribing, a complex, multilevel behavior that is important for ensuring patient safety, seven domains met the criteria for perceived “relevance” and could be targeted in interventions to improve prescribing. In addition, in a departure from previous TDF practice, through a critical appraisal of the data, we identified that two domains were problematic and should also be targeted, despite being coded as “not relevant” in the previous stage. These were “beliefs about consequences” and “beliefs about capabilities.” In light of the high error rate found in a related prospective observational study (PROTECT manuscript in preparation), beliefs that prescribing errors were unlikely to have consequences for patients and that trainee doctors are capable of prescribing without error should also be targeted in an intervention. This study is the first to suggest a critical approach to domain identification for intervention development and the first to propose theoretical elaborations and interrelationships between domains based on interview findings. Finally, the study is the first to use this theoretical framework in relation to prescribing errors, and the findings provide an evidence base for complex intervention design.

## Competing interests

The authors declare that they have no competing interests.

## Authors’ contributions

EMD developed the topic guide, conducted the interviews, analyzed the data, and led the writing of the paper. JJF contributed to the design of the study, critiqued the topic guide, analyzed data, reviewed results, and contributed to the writing of the manuscript. MJ contributed to the design of the study, critiqued the topic guide, and provided advice on analysis. PD, SM, JM, GAM, and DJW contributed to the design of the study and interviewee recruitment and reviewed various drafts of the manuscript. SR codeveloped the research, critiqued the topic guide, and contributed to review of results and writing of the manuscript. CR critiqued the topic guide, contributed to review of results, and reviewed various drafts of the manuscript. CB designed and was PI for the research, contributed to the development of the topic guide, commented on pilot interviews, and critiqued successive drafts of the paper. All authors read and approved the final manuscript.

## Supplementary Material

Additional file 1 Interview topic guide. Click here for file
