# Supplementary material for: Learning curves, taking instructions, and patient safety: using a theoretical domains framework in an interview study to investigate prescribing errors among trainee doctors
Source: Implement Sci. 2012 Sep 11;7:86. doi: 10.1186/1748-5908-7-86 (PMC3546877; doi:10.1186/1748-5908-7-86)
Supplement: Additional file 1 — Interview topic guide. [file 1748-5908-7-86-S1.doc]

**Additional file 1**: Specific beliefs from thematic coding

| **Specific Beliefs** | **Illustrative Quotes** |
| --- | --- |
| **LEARNING CURVES** | |
| - **Influence of gaining clinical experience** | |
| My perceptions about my own prescribing have changed with experience | To begin with as an F1 you’re very cautious about prescribing and I’ll always remember that first time when I prescribed paracetamol and I’m thinking am I making a mistake. Whereas now I can someone paracetamol and think to myself what I’ve done’s right. So there is that difference with the experience and the confidence that you gain. INTERVIEWEE 3 (F2) |
| When I have more experience I consult reference sources less | I remember at the start I would have to ask every time I was gonna prescribe something. I just wanted to check that it was fine but now you kinda do things day to day and you kind of get used to what you should be prescribing. INTERVIEWEE 12 (F1) |
| Less experience means I may be more likely to make an error | I think you are more likely to make an error when you are more junior because you don’t have the experience. INTERVIEWEE 11 (F2) |
| More experience means I may be more likely to make an error | I think with experience you obviously know more about medications and why you give them, but then, with experience you also, people may also get umm, careless … Careless as in like people might not bother to check with the BNF if they’re either like half sure but then they think that they’re sure but in fact they’re not actually. INTERVIEWEE 4 (F1) |
| - **Knowledge required: procedural/clinical** | |
| I need to know professional norms for writing prescriptions in order to prescribe without error | Just obviously the correct way to fill out a kardex and certain things you’re not supposed to… Certain abbreviations you can use, like for instance you’re supposed to always write oral or not p.o., but to be honest loads of people write o and p.o. and all that stuff. INTERVIEWEE 13 (F1) |
| I need to know about guidelines and protocols to prescribe without error | Well you need to know what local guidelines are and we have local guidelines that are not meant to be used like PRN and things like that and using circles for hourly and things like that so local guidance on how to write prescriptions and things like ... there is always a thing that your writing has to legible and you need to know what drug you are prescribing and what you are prescribing it for and what patient it’s for. INTERVIEWEE 8 (F2) |
| I’m not always aware what protocols are in place | That’s about it really, I’m not really aware of any… I haven’t seen any written protocols but that doesn’t mean to say that they don’t exist, probably just that I haven’t been aware of them. INTERVIEWEE 10 (F1) |
| **TAKING INSTRUCTIONS** | |
| - **Inter-professional responsibility** | |
| The nurses are good at picking up errors | If there is any reason that there is a problem with the prescription the nurses will mark it on the Kardex and will come to you afterwards and say can you check this, was this, is this definitely what you want to give. Sometimes if you have more junior nurses may not know that there is an error with that prescription they might still give the prescription. INTERVIEWEE 11(F2) |
| The pharmacist checks my prescriptions for errors (in some wards only) | we’ve got like pharmacists who do are meant to sort of double check all the Kardexes and they’ll come up to you and say can you just change this if there’s anything that’s wrong but it does come down if your signatures against the drug then it is you know you’re the one that’s gonna be liable if there’s any serious consequences from it so. INTERVIEWEE 5 (F1) |
| - **Influence of others** | |
| Senior colleagues influence my prescribing behaviour | And, you now, the nature of the job is that you, when a consultant tells you ‘Go and prescribe this amount of drug for this person’, you’re not going to turn round to them and say ‘Why?’. INTERVIEWEE 2 (F1) |
| Pharmacists influence my prescribing behaviour | the pharmacists, as I say, are really approachable and if there’s ever been any issues with stuff I don’t understand or if they’ve asked me to do something that I’m not entirely sure why, I’ll always ask them about it and they’re always more than happy to teach me about it. INTERVIEWEE 10 (F1) |
| Nurses influence my prescribing behaviour | even though the nurses are not allowed to prescribe, they will come and tell you this is what I want you to prescribe and generally it’s good to follow their advice because they know. INTERVIEWEE 11 (F2) |
| Everything I write on a prescription will have been told to me by a senior colleague | I certainly wouldn’t go against what a senior registrar is advising, even though it’s my signature at the end of the day. INTERVIEWEE 1 (F1) |
| - **Influence of medical speciality** | |
| Support is greater when working on specialist wards | Because renal medicine is quite specialist, a lot of the specialist drugs, I’d never heard of before and am not prescribing for, which is why they’ve got a pharmacist with a special interest in renal pharmacology down here, so she’s always on hand to ask anything. There’s always seniors as well, because it’s so specialist down here, to ask them, if you have any queries over prescribing. INTERVIEWEE 5 (F1) |
| **PATIENT SAFETY** | |
| - **Confidence about prescribing without error** | |
| I’m confident I don’t make errors when prescribing | Yeah, I’m quite confident that I’m quite a safe prescriber, to what extent I don’t know. INTERVIEWEE 13 (F1) |
| - **Error outcomes** | |
| If I make an error it will be picked up my someone else | I think sometimes some of the things you might forget are simple things that probably wouldn’t actually result in an error, like not giving a time for it to be given. Well, I suppose that’s an error though isn’t it, because then something might be missed? But hopefully it would be flagged up or the nurse would notice it and get somebody to put a time in. INTERVIEWEE 13 (F1)  They’re kind of simple errors and also actually the patient themselves will tell you no I take that at night, or no I take that at lunchtimes so you can just go back and revise that but you do have to keep an eye on that. INTERVIEWEE 11 (F2) |
| If I make an error nothing may happen | There’s always the possibility that nothing could happen and that it wouldn’t be spotted in which case nobody would be any the wiser. INTERVIEWEE 3 (F2) |
| If I make an error it may not have any effect on the patient | If you give it to them and even if they won’t sleep, it’s not really an error, it’s not going to have really, really massive negative impact on the patient but it will cause them some discomfort. INTERVIEWEE 11 (F2) |
| If I make a prescribing error it can cause harm to the patient | if the patient’s getting the wrong medication, they’re getting an increased dose or potentially a different drug altogether, then there’s going to be an adverse drug reaction., potentially fatal, quite a serious thing. INTERVIEWEE 1 (F1) |
| If I make a prescribing error it can result in negative outcomes for myself | you could get struck off and the most severe you know serious consequences or taken to court but even if it was something smaller your you know it knocks you’re confidence if you got pulled up for doing something that you know where even if It were a minor consequence for a patient I suppose you know its that kinda like patients would lose their trust in you … then if it was me I wouldn’t want the same doctor sort of prescribing things for me again. INTERVIEWEE 5 (F1) |
| - **Situations associated with errors** | |
| If I am distracted when I’m prescribing I’m more likely to make an error | I think less mistakes would happen if you could sit in a quiet room and write out the charts quietly rather than on a busy ward with people coming up to you and telling you that so and so’s blood pressure is such and such, or that you need to go and do this, that or the other, or can you… It’s just if you’re constantly being interrupted then… INTERVIEWEE 10 (F1) |
| If I am under time pressure when I’m prescribing I’m more likely to make an error | Just the sheer amount of work that you’ve got to do. If you’re rushing a prescription then errors are going to creep in then. INTERVIEWEE 1 (F1) |
| - **Strategies to reduce errors** | |
| Using reference sources helps me to prescribe without making an error | I think I was prepared to look everything up even if it took twice as long just to make sure that I was writing it correct and it did take me a while to know doses of different things and painkillers like Tramadol and things like that. INTERVIEWEE 8 (F2) |
| Having easily available guidance at the point of prescribing medications would reduce errors | The protocols as I say for the antibiotics are on the walls in every doctors room so you know you don’t need to just remember off the top of your head I mean you should and you do as time goes on you remember common antibiotics but if you’re unsure I mean it’s just a case of just like popping and it’s there on the wall and it’s got the dose and it’s got the times and stuff and you know the duration that you would keep them on it for and everything. INTERVIEWEE 5 (F1) |
| Having greater pharmacy support would reduce prescribing errors | In lots of ways ward based teaching would be really useful, if the pharmacist… I think they do do this actually on some of the wards, the pharmacists do ward based teaching, but I’ve not had it on any of the wards that I’ve been on. I think that might be quite useful. INTERVIEWEE 10 (F1) |
